# Classification of Organic and Conventional Vegetables Using Machine Learning: A Case Study of Brinjal, Chili and Tomato

**DOI:** 10.3390/foods12061168

**Published:** 2023-03-09

**Authors:** Sowmya Natarajan, Vijayakumar Ponnusamy

**Affiliations:** Department of Electronics & Communication, SRM Institute of Science and Technology, Kattankulathur, Chennai 603203, India

**Keywords:** organic food, organic vegetables, conventional vegetables, machine learning, multispectral spectroscopy, neural network, ant colony algorithm, random forest algorithm

## Abstract

Growing organic food is becoming a challenging task with increasing demand. Food fraud activity has increased considerably with the increase in population growth. Consumers cannot visually distinguish between conventional and organically grown food products. Spectroscopic methodologies are presented to identify chemicals in food, thereby identifying organic and conventional food. Such spectroscopic techniques are laboratory-based, take more time to produce an outcome, and are costlier. Thus, this research designed a portable, low-cost multispectral sensor system to discriminate between organic and conventional vegetables. The designed multispectral sensor system uses a wavelength range (410 nm–940 nm) that includes three bands, namely visible (VIS), ultraviolet (UV) and near-infrared (NIR) spectra, to enhance the accuracy of detection. Tomato, brinjal and green chili samples are employed for the experiment. The organic and conventional discrimination problem is formulated as a classification problem and solved through random forest (RF) and neural network (NN) models, which achieve 92% and 89% accuracy, respectively. A two-stage enhancement mechanism is proposed to improve accuracy. In the first stage, the fuzzy logic mechanism generates additional feature sets. Ant colony optimization (ACO) algorithm-based parameter tuning and feature selection are employed in the second stage to enhance accuracy further. This two-stage improvement mechanism results in 100% accuracy in discriminating between organic and conventional vegetable samples. The detected adulterant is displayed on a web page through an IoT-developed application module to be accessed from anywhere.

## 1. Introduction

Currently, a small portion of around 1–2% of total agricultural land accounts for organic farming in India [1]. Still, organic farming is in the growth stage and is developing well. Both domestically and internationally, organic products in markets are increasing. However, some challenges exist in developing and commercializing organic products due to the lack of certification, limited market access, investment and consumer awareness. Environmental and health concerns drive the demand for organic products to gain maximum profit, although they are more expensive than conventional products. Due to the lack of production, limited supply and higher cost, fraudulent activities have increased. False labeling, chemical spraying on products to look fresh and false marketing are common problems that undermine consumer confidence in the authentication of organic products. As demand for organic products grows, developing a system model that helps identify organic products is required. It is convenient to undergo spectroscopic measurements such as near-infrared (NIR), Fourier transform infrared (FT-IR), mid-infrared (MIR), terahertz and nuclear magnetic resonance (NMR) to distinguish between organic and conventional food products. Spectroscopic measurements are used to measure light absorbance at different wavelengths, which helps measure the concentration of substances in a material.

Beer–Lambert’s law defines a linear relationship between the absorbance of a substance, molar absorption coefficient, optical coefficient and concentration, given by

Ω = ε χl(1)

where Ω—absorbance; ε—molar absorptivity (m^−1^cm^−1^); χ—concentration of substances; l—length of sample.

The amount of light reflected after absorption by organic and conventional vegetables is utilized for distinguishing between the conventional and organic constituent elements present in the vegetables.

A few literature works have been reported to detect organic and conventional food items using spectroscopic methods.

NIR spectroscopic methods were used to evaluate the differentiation between conventional and organic apples [2]. The results show significant differences between conventional and organic apples according to sugar–acid ratio, volume and color. According to volume and color, the outer appearance difference achieves a robust significant correlation of analysis of variance (ANOVA) (*p* < 0.0001), and the sugar–acid ratio obtains a weak significant correlation of ANOVA *p* = 0.04 between organic and conventional apple samples.

A review work focused on identifying organic and conventional vegetables and fruits. Electron paramagnetic resonance spectrometry (EPR) [3] was used to determine the consecutive measurement of nitrate and nitrite ions in vegetables and fruits. The quantity of nitrite and nitrate ions in 18 different vegetables and fruits was compared between those homegrown using organic fertilizers and local markets. The highest concentration of nitrate ions for vegetables was 38.9 mg/kg for homegrown products and 451.6 mg/kg for local market products. The experimental analysis revealed that 0.45 mg/kg nitrite ion concentration are found in homegrown beetroot and 1.54 mg/kg nitrite ion concentration for local market purchased beetroot samples. The lowest nitrate concentration is found in potato (17.6 mg/kg homegrown and 40.2 mg/kg local market), and nitrite ion achieves 0 mg/kg for homegrown potato and 0.66 mg/kg for local market potato. Biosensors and chemometric analysis play a significant role in determining disease diagnosis [4,5].

A study [6] was conducted to determine the influence of agronomic practice on antioxidant vitamins and phenolics concentration in the yellow plums. Organic yellow plums were grown on three different soils: *Trifolium*, tilled soil and soil covered with natural meadow conventional plums are grown on tilled soil. Proximate analysis revealed that antioxidant vitamins and phenolic compounds are at the maximum concentration in organically grown plums.

In farming field trials [7], Chinese yam was used to discriminate between conventional and organic. Manganese (Mn), chromium (Cr), Se, Na, As, δD and δ15N are crucial variables that are used to discriminate between yam samples. Mn, Cr, Se, Na and As are essential to determine the presence of nutrients in Chinese yam. Stable isotopes were used to trace the toxic substances in the samples. Mass spectrometry analyzed the contaminant and chemical component activity of yam samples. The random forest (RF) classifier achieved 97.3% classification accuracy for organic and conventional yam discrimination.

The use of dyeing agents on food items to make them look fresh and prolong the storage period was analyzed, as this practice harms individuals. ^1^H NMR relaxometry [8] was deployed to determine the presence of Sudan dye, malachite green and copper sulfate on red chili, peas and okra. Homegrown vegetable samples were utilized by spraying the dyes and soaking the vegetables in different solvents. The results show that ^1^H NMR relaxometry can quantify the presence of dyes to the lowest value of 1 g/L (0.1%).

Table 1 summarizes the various spectroscopic methods for discriminating between organic and conventional vegetables and fruits. Both destructive and nondestructive methods are deployed to evaluate the compositional elements and classification. Machine learning algorithms are deployed for the analysis of spectral information. A maximum accuracy of 100% and a minimum accuracy of 85.9% are reported from the destructive spectroscopic analysis for the discrimination of conventional and organic vegetables and fruits.

NIR spectroscopic measurement [9] with three pattern recognition methods, namely PLS-DA, KNN and moving-window PLS-DA, was employed for classifying apple sample varieties. Four apple varieties were classified: Fuji, new Jonagold, Red Star and Ralls Janet. The results indicate that moving-window PLS-DA delivers an accuracy of 98% more than KNN.

A computer vision-based sensor system [10] was presented using spectroscopic image data to discriminate between conventional and organic apples. It involves five pattern recognition algorithms such as support vector machine (SVM), k-nearest neighbor (K-NN), partial least-squares discriminant analysis (PLS-DA), kernel PLS-DA and locally weighted partial least-squares classifier (LW-PLSC). A maximum classification accuracy of 94% was obtained by the locally weighted partial least-squares classifier algorithm. A subsequent work explored the NIR spectroscopy system for distinguishing between organic and conventional Gala apples [11]. The spectral data were preprocessed, baseline-corrected and normalized for classification by PLS-DA. The results show that a classification accuracy of 98% is achieved for discriminating the Gala apple samples.

To differentiate between conventional and organic tomatoes, ^1^H proton nuclear magnetic resonance (^1^H NMR), isotope ratio mass spectrometry (IRMS) and MIR spectroscopy were utilized [12]. The spectral data were processed with linear discriminant analysis (LDA), principal component analysis (PCA) and PLS-DA algorithms. The individual spectroscopy accuracy results are as follows: NMR—91.3%–100%, MIR—75%–91.7%. Of the validation results, 100% were obtained for fused data (^1^H NMR+ IRMS+ MIR) with the LDA method.

The authors of [13] carried out a study to discriminate between organic and conventional pineapples by extracting the total soluble solids contents. The portable NIR spectroscopic technique combined with machine learning (ML) algorithms such as KNN, LDA, PCA, PLS-DA and multiplicative scatter correction–principal component analysis–linear discriminant analysis (MSC-PCA-LDA) was used to classify organic and conventional pineapple samples. Among the above models, MSC-PCA-LDA provides 100% classification accuracy based on the total soluble solids (TSS) present in pineapples. Two more NIR spectroscopy techniques [14,15] were employed to differentiate between conventional and organic food materials by examining the soluble solid content (SSC) in fruits, vegetables and dairy products.

A nondestructive spectroscopic system [16] was used to predict the SSC of apple samples. An optical setup was used to obtain the multispectral data from the samples. A multiple linear regression model analyzed and predicted the root mean square error at 0.861.

Another work [17] classified whether the diuron herbicide content is within the maximum residual limit (0.2 ppm) or not on intact olives using NIR spectroscopic technology. The spectral validation dataset achieved 85.9% classification accuracy for binary class of C1: diuron level > 0.2 ppm and C2: ≤ 0.2 ppm.

Most of the reported spectroscopic analyses are based on destructive sample analysis, which is expensive, laborious, bulky and unsuitable for the real-time analysis of organic and conventional discrimination. Portable, low-cost spectroscopic sensor systems [18] were previously designed and applied in milk adulteration detection; however, no attempt has been made to discriminate between organic and conventional vegetables. A real-time, cost-effective and reliable detection mechanism is required to detect organic vegetables and fruits. To overcome these flaws, this research work focuses on the following:

- Our proposed work includes the design and construction of a nondestructive, portable, rapid-analysis multispectral sensor system to discriminate between conventional and organic vegetable samples using random forest and neural network algorithms.
- A multispectral sensor system is designed with three bands of NIR, UV and visible to enable reliable detection.
- In practical use of the sensor system, the accuracy of detection is challenged by the influencing factors of the angle of placement of the sensor on the sample, the distance between the sensor and sample, the presence of ambient light, the size of the sample, variation in the color texture of the sample. Fuzzy-logic-based feature generation is employed to scope-up such variable factors.
- The ant colony algorithm is utilized for feature selection and parameter tuning to improve detection accuracy in the presence of variable factors and reduce the response time.

The remainder of this article is arranged as follows: Section 2 presents the materials and methods, the development of an AI-enabled multispectral sensor system, the design of a fuzzy engine for feature generation, feature selection using the ACO algorithm, and random forest and neural network models for discriminating between organic and conventional vegetable samples, followed by a discussion of sample preparation and data acquisition. Section 3 presents the results and discussion of the histogram analysis and three-band spectral responses, followed by ant colony, random forest and neural network algorithm results. Finally, Section 4 concludes the work with the future scope.

## 2. Materials and Methods

This section describes the multispectral sensor system design. Data acquisition, fuzzy logic feature generation, optimized feature selection, and parameter tuning by ACO algorithm are delineated. Random forest and neural network models are presented to classify organic and conventional vegetable samples.

### 2.1. Multispectral Sensor System Model

**Figure 1:**
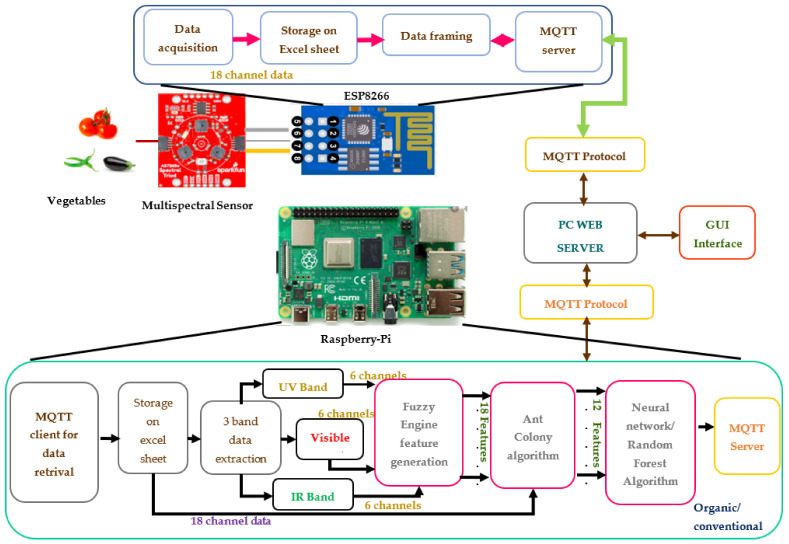
Block diagram of artificial intelligence (AI)-enabled organic and conventional discrimination using IoT system.

The designed and developed prototype for organic and conventional vegetable classification is given in Figure 1. It comprises the hardware components of the Triad Spectroscopic sensor system AS7265X, ESP8266 and Raspberry Pi. Its software component uses a fuzzy engine for feature generation.An ACO algorithm software module for optimizing parameter and feature selection. Neural network and random forest machine learning modules are used for the classification of organic and conventional samples, an MQTT client and server module are used for moving the data to a PC server, and a PC web server module with a GUI is used to display the result.

The Triad sensor holds three optical sensors: (i) AS72651, (ii) AS72652 and (iii) AS72653. Each sensor has 6 optical filters independently and a total of 18 channels with wavelengths ranging from 410 nm to 940 nm. The AS72651 is a master and holds ultraviolet (UV) ranges from 610 nm to 860 nm. AS72652 and AS72653 act as slave sensors that can sense near-infrared and visible regions from 560 nm to 940 nm [19,20]. The sensor has 3 integrated LED light sources of white LED with a luminescence of 5700 k, a UV LED emitting a wavelength of 405 nm and an IR LED with a light emission of 875 nm. The field of view of the sensor is ±20.5°.

All channel data in the Triad sensor represent the received photon count β reflected after absorption from the measurement surface. The received photon counts β are defined by

β = α − γ*ε χ l(2)

where α—intensity of incident LED light source; γ—amount of light incident on sample; ε χ l—number of photons absorbed in samples; l—sample length; ε—molar absorptivity; χ—concentration of substance.

ESP8266 is connected to the sensor system through a QWIIC cable. It acquires the data in three bands (visible and NIR) with 18 channels through the data streamer of an Excel sheet. The software module for data acquisition and formatting with the message queuing telemetry transport (MQTT) protocol and a web client is deployed on ESP8266. Using the MQTT protocol, the web client communicates and stores the 18-channel data to the web server.

In the system, the data acquisition module is separated from the data processing module to support scalability. ESP8266 is used for data acquisition alone, and Raspberry Pi is used for data processing to run machine learning algorithms. The Raspberry Pi processor runs a software module for data splitting, fuzzy engine, ACO and the machine learning algorithm of the neural network and random forest algorithm. The Raspberry Pi processor obtains the 18-channel data stored in the web server using the MQTT protocol. The 18-channel data are split into three-band data for further processing. The fuzzy logic engine is designed to generate a new 18 fuzzy feature set using three-band split data. The ACO optimization algorithm selects the 12 best features from the 18 fuzzy features. These selected features are used for classification using the artificial intelligence (AI) machine learning algorithm of random forest (RF) ensemble learning and the neural network (NN) algorithm. The random forest and neural network models are designed for the binary classification of organic and conventional vegetables. This research aims to fulfill the sharing of discrimination information of conventional and organic vegetables with the end-user community. The Internet of Things (IoT) is used for this purpose. IoT technology connects things to the internet to enable output response accessibility from anywhere. Therefore, the sensor system is IoT-enabled to only publish the result on the internet for access to the public community. A web page is designed with a user-friendly graphical user interface (GUI) to display the result and acquired data. A password-based authentication system is developed to implement a security mechanism for access.

#### 2.1.1. Fuzzy Model for Feature Generation

Fuzzy logic is designed with a set of rules to solve decision-making problems when the data are fuzzy in nature [20]. Fuzzy logic consists of three process stages: fuzzifier, intelligence and defuzzifier. Here, fuzzification and defuzzification are applied to generate a new feature set. In our spectral sensor, variable factors that affect the sensor response are (i) the distance between the sensor and the sample, (ii) the focus angle of the sensor and (iii) the variation in the size of the samples. Such factors introduce fuzziness in the acquired data set. Fuzzy-logic-based new feature generation is used to handle this fuzziness. Fuzziness in a dataset is determined using the membership function. In this system, the triangle membership function is used.

The triangle fuzzy membership function is defined as

(3) $$\mathrm{Triangle}\;(x:\;a;\;b;\;c)=f(x)=\left\{\begin{array}{c} \frac{x-a}{b-a\;};a\leq x\leq b\\ \frac{c-x}{c-b};b\leq x\leq c\\ 0;\mathrm{otherwise} \end{array}\right.$$

where a—lowest edge point of triangle; b—top point of triangle; c—highest edge point of triangle; x—input variable.

Table 2 gives the input membership function a,b,c triangular point values used to fuzzify the sensor values. Defuzzification is applied to generate a new feature set. After the fuzzifying and fuzzy inference process, defuzzification is applied to generate new feature data.

In this work, a centroid-based defuzzification is applied. The centroid method to compute the output y_defu_ for fuzzy input x is defined as

(4) $$y_{defu}=\frac{\int \mu_{i}\left(x\right)xdx}{\int \mu_{i}\left(x\right)xdx}$$

where *μ_i_* (*x*)—aggregated membership function; *y_defu_*—membership degree output.

Table 3 defines the output membership functions associated with converting the fuzzy feature value into a crisp value. The 18-channel new feature is generated using fuzzy logic, which is applied to the ACO algorithm for the feature selection mechanism. The feature selection of the ACO algorithm is discussed in the next section.

#### 2.1.2. Feature Selection Using Ant Colony Algorithm

Our proposed system involves 18 channels of data, which are acquired at 3 different spectral bands. Using all 18 channels with 3 bands may overfit the machine learning model; therefore, feature selection is essential for this work. It is observed that the spectral data show significant variation with respect to ambient light conditions, the distance between the sensor and sample, the focus angle of the sensor to the sample, the dimension of the samples and variation in the sample color texture. New fuzzy feature generation and feature selection processes are carried out to scope-up such variation factors and improve classification accuracy. Usually, evolutionary algorithms such as genetic and ACO algorithms are used for optimized parameters and feature selection [21]. This work uses the ACO algorithm to search for an optimal feature subset. The ACO algorithm generally uses the probabilistic method to solve optimal path-finding problems such as the behavior of ants searching for food. The ACO algorithm builds a graph with n features as nodes and k ants, which are initially assigned in $V_{list}^{k}$ records as the ants visit nodes. The pheromone δ_ij_ (0) is assigned an initial value of 0. In this work, the optimal feature selection problem is formulated as an optimal path-finding problem and solved by an ACO algorithm.

Sometimes, the ACO algorithm fails to provide considerable performance and produces reduced feature dimension due to low (poor) diversity in selecting the features.

Two-stage updating is performed for pheromone updating to improve the performance of an algorithm under the above case. In the first stage, updating is intended to increase the particular path pheromone value, and in the second stage, it is used to prevent falling in the local optimum. By performing this update, the optimal feature subset can be sorted, the accuracy of the classification can be enhanced, and feature subset dimensions can be reduced.

The algorithm of ACO follows the following steps to select optimal features.

Step 1: Generate random pheromone, initial outcome and ant k = 0;

Step 2: In iteration repeat.

Ant selects the next node to move based on the pheromone concentration. The path transfer probability $\psi_{ij}^{k}\left(t\right)$ in which the ant moves from feature i to j node for iteration t = 0 is defined as

(5) $$\psi_{ij}^{k}\left(t\right)=\left\{\begin{array}{c} \frac{\delta_{ij}^{\Gamma_{i}}\left(t\right)h_{ij}^{\epsilon_{h}}\;\left(t\right)}{\sum_{s\notin V_{list}^{k}}\delta_{is}^{\Gamma_{i}}}(t)\;h_{is}^{\epsilon_{h}}\left(t\right)\;\;\;\;i,\;j\notin V_{list}^{k}\\ 0,\;\;\;\;\;\;else \end{array}\right.$$

In general, *h_ij_*—heuristic function (1/*ed_ij_*); *ed_ij_*—Euclidean distance.

However, for the two-stage pheromone updating process, *h_ij_* can be redefined as follows

(6) $${h_{\mathrm{ij}}}_{\;}=\frac{TPR}{ed_{ij}}$$

where *TPR*—true-positive rate, which is defined as

(7) $$TPR=\frac{TP}{TP+FN}$$

where *TP*—true positive; *FN*—false negative.

*δ_ij_*(*t*)—path pheromone concentration from *i*th to *j*th features during t iterations;

Γ*_i_*,$\;\epsilon_{h}$—information and expectation heuristic factors used to assign weights.

After the traversal of ants based on the probability of Equation (5), the information concentration (pheromone) is updated as,

(8) $$\delta_{\mathrm{ij}}=(1-p)\;\delta_{\mathrm{ij}}+p\;\sum_{k=1}^{m}△\delta_{ij}^{k}$$

p—weight coefficient (0< *p* < 1); △$\delta_{ij}^{k}$—pheromone path increments between the feature i and j node of k^th^ ant is given by

(9) $$\Delta \delta_{ij}^{k}=\left\{\begin{array}{c} \frac{\Pi }{l_{k}},\left(i,j\right)\in \mathrm{path}\;\mathrm{of}\;k\\ 0,\;\;\;\;\;\;\;\;\;\;\;\mathrm{else} \end{array}\right.$$

Π—constant; l_k_—path length of *k*th ant in traversal.

Step 3: Select a subfeature set and parameters for machine learning models. Calculate the fitness function for feature subset and classification accuracy.

Using the fitness function [21].

Λ = ξ FPR + (1 − ξ) (S_fd_ + S_p_)/S_n_ (10)

ξ—balancing weight between feature dimension and classification performance;

S_n_—dimension of the whole set traversed by ants;

S_fd_—selected feature subset;

S_p_—selected parameter set;

FPR—false-positive rate, which is defined as

(11) $$\mathrm{FPR}=\frac{FP}{TN+FP}$$

*FP*—false positive;

*TN*—true negative.

Step 4: Check whether the maximum accuracy reached if met, stop and take the currently selected subfeature as the optimal feature set and the selected parameter set as the optimal parameter.

If not, perform two-stage pheromone updating in Steps 5 and 6.

Step 5: Stage 1. The total path traversed by ants for iteration t, updated for pheromone concentration

(12) $$\delta_{ij}^{1}\leftarrow (1\;-\;p)\delta_{\mathrm{ij}}+\kappa \;\sum_{k=1}^{m}△\delta_{ij}^{k}$$

k—ant number; m—total number ant colony in iteration; κ—pheromone concentration attenuation coefficient.

△$\delta_{ij}^{k}$(t)—pheromone concentration released by ant k on the path (I, j) for t iteration, given by

(13) $$\Delta \delta_{ij\;}^{k}\left(t\right)=\left\{\begin{array}{c} \theta CF\left(f^{k}\left(t\right)\right)+\frac{1-\theta }{\left|f^{k}\left(t\right)\right|},\left(i,j\right)\in f^{k}\left(t\right)\\ 0,\;\;\;\;\;\;\;\;\;\;\;\;\;\;\;\;\;\mathrm{else} \end{array}\right.$$

f^k^ (t)—feature subset is chosen by ant k in t iteration;

| f^k^ (t)| length of feature subset;

$\mathrm{CF}\left(f^{k}\left(t\right)\right)-an$ index measuring classifier performance.

(14) $$\mathrm{CF}\left(f^{k}\left(t\right)\right)=\frac{TP+TN}{TP+FP+TN+FN}$$

where *TP*—true positive; *TN*—true negative; *FP*—false positive; *FN*—false negative.

Step 6: Stage 2. Pheromone update

The pheromone change value of *t*th iteration of the best ant can be defined as

(15) $$\Delta \delta_{ij\;}^{bs}\left(t\right)=\left\{\begin{array}{c} ŋ△\delta_{ij\;}^{bs}\left(t\right);\;\mathrm{arc}\;\left(i,\;j\right)\;\epsilon \;\mathrm{path}\;\mathrm{of}\;\mathrm{best}\;\mathrm{ant}\;\\ 0;\;\;\;\;\;\;\;\;\;\;\;\;\;\;\;\;\;\;\;\mathrm{else} \end{array}\right.$$

*bs*—optimal solution created in iteration by total ants; ŋ—pheromone concentration for special paths.

Pheromone addition of the nearest ant and the best ant is given as

(16) $$\Delta \delta_{ij\;}^{near}\left(t\right)=\left\{\begin{array}{c} \left(1-ŋ\right)△\delta_{ij\;}^{bs},nearest\;path\;of\;best\;ant\\ 0,\;\;\;\;\;\;\;\;\;\;\;\;\;\;\;\;\;\;\;\;\;\;\;else \end{array}\right.$$

The Stage 2 pheromone concentration update can be defined in Equation (19) using the best ant and nearest ant addition values

(17) $$\delta_{ij\;}^{2}\leftarrow \;\delta_{ij\;}^{1}+\Delta \delta_{ij\;}^{bs}\left(t\right)+\Delta \delta_{ij\;}^{near}\left(t\right)$$

Step 7: Increase the number of ant k = k + 1, and go to Step 2

The optimally selected feature set from the outcome of the ant colony algorithm is used as input to the random forest and neural network machine learning algorithm to perform accurate classification with reduced time complexity. The details of the random forest algorithm classification are discussed in the next section.

#### 2.1.3. Random Forest Algorithm

A random forest algorithm is constructed with decision trees on different spectral samples and chooses the majority vote for the final decision which is presented in Algorithm 1. It uses ensemble learning, combining many classifiers to solve complex problems. The symbols used for the random forest algorithm can be defined as follows [22]

N_P_ ← partition; N_d_—number of the decision trees in a random forest; R_np_—ratio of negative and positive cases;

Ts—sampling threshold; R_nn_—radius of nearest neighbor; bootstrap works with respect to the sampling process;

M_ot_—optimal model; Acc_M_—accuracy; P_ot_ ← optimal parameter.
**Algorithm 1:** Ensemble Random Forest algorithmInput: best feature set from ant colony S_fd_, parameters: Ts, N_P_, R_np_, N_d_, R_nn_, function ant_call () for ant colony,Output: Optimal model1. Use optimal feature set and parameters from ACO algorithm as the input dataset2. Ensemble—RF (S_fd_, Ts, N_P_, R_np_, N_d_, R_nn_)3. For n < −1 to N_P_ do     a. (Train, test) ← random split (Sfd, Ts)     b. Split ← bootstrap (train, Rnp, Rnm)     c. Model ← Random Forest. Train (split, Nd)     d. Score ← evaluate (model, test)     e. Out[n] ← (model, score)      End for      $f.\;[\mathrm{Optimal}\;\mathrm{model}\;\mathrm{Mot},\;\mathrm{accuracy},\;CF\left(f^{k}\left(t\right)\right)$] = Call voter for optimal model selection]     g. Call ant_call ( ) function for parameter tuning and feature selection.     $[P_{\mathrm{ot}},\;S_{\mathrm{fd}}]=\mathrm{ant}\_\mathrm{call}\;(M_{\mathrm{ot}},\;CF\left(f^{k}\left(t\right)\right)$)     h. If (the actual performance is less than the target performance),     then go to Step 1      Else go to Step 4     End if4. Return out

The neural network is another machine learning algorithm utilized in the proposed method to test classification accuracy, which is discussed in the next section.

#### 2.1.4. Neural Network Architecture

**Figure 2:**
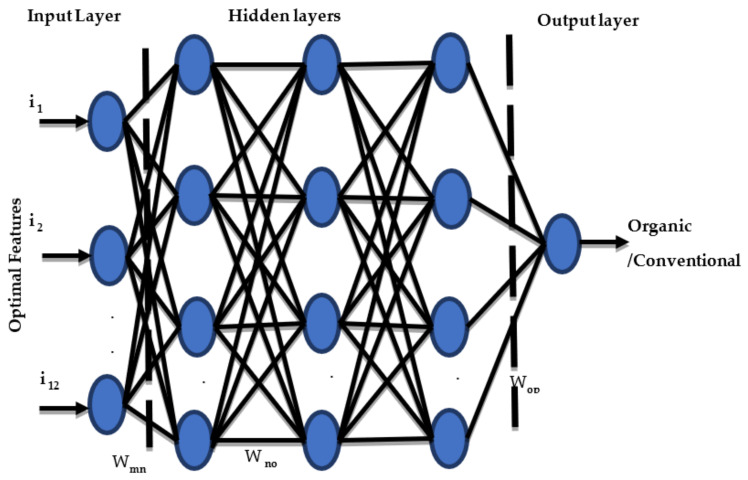
Neural network architecture.

Figure 2 illustrates the neural network’s architecture with 12 optimal features, i_1_, i_2_, i_3_,…, i_12_, which represents the input layer neurons trailed by ten hidden layers, and a single neuron is designed for binary class classification at the output layer. W_mn_ constitutes weights between the input and hidden layers, W_no_ constitutes weights between the interconnected hidden layers, and W_op_ constitutes weights between the last hidden layer. The output layer calculates the error value at the output by comparing the predicted and target values. This error value propagates in backpropagation from the output to the input layer. The weight values are adjusted with the gradient descent method to minimize the error value and backpropagate the errors.

Cross-entropy is employed as a loss function, which measures the error value between the target and predicted value, and it is represented by

(18) $$\mathrm{CE}=-\sum_{i=1}^{n}\;\Theta log\left(r_{i}\right)$$

where Θ—true value; r_i_—sigmoidal probability of *i*th class. Cross-entropy is used to reach the minimum possible error, and in turn, the weight value is updated.

The sigmoidal activation function is used in the output layer for binary classification. It is given by

(19) $$F\;(u)=1/(1+e^{-v}))$$

where v—input; F(u) = output.

### 2.2. Sample Preparation

This section describes the land and sample preparation procedure, followed by multispectral spectral data collection. Land preparation is one of the key elements in agriculture sample cultivation, especially when preparing vegetable samples for organic and conventional discrimination. Nutrition component analysis also discriminates between conventional and organic vegetables and fruits [23]. The organic and conventional vegetable samples were grown in the same red soil in different areas with a proper water supply.

**Figure 3:**
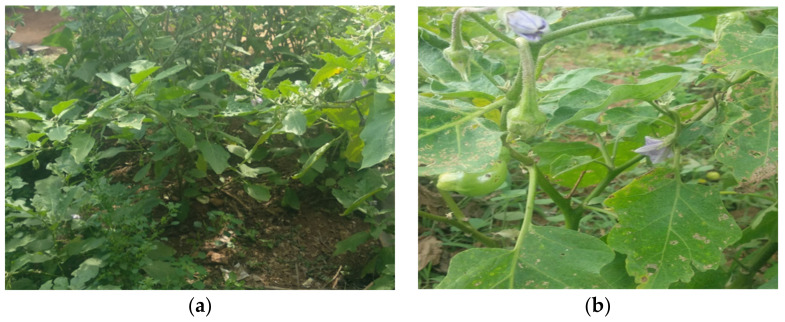
(**a**) Organic plant for brinjal cultivation; (**b**) conventional brinjal plant.

Figure 3a,b depicts a visual representation of organic- and conventional-grown brinjal plants. Soil fertility plays a significant role in growing organic food products. The soil should have enough nutrient and mineral supply for plant growth [24]. The red soil land is divided into two separate 10 × 10 square feet areas, one for growing organic and another for conventional vegetable samples for cultivation. Both areas are maintained at a distance of 15 m from each other during cultivation. The organic land is sprayed with organic fertilizers such as cow dung and goat dung. The fertilizers used for the conventional area are diammonium phosphate (DAP) 18:46 and single super phosphate (SSP).

In total, 70 vegetable samples were obtained from the farm. One or two samples from each were eliminated due to damaged, diseased and spoiled conditions. Out of 25 counts of tomato (red color), 11 were organic and 13 were conventional. Similarly, in brinjal (purple), 12 were organic and 10 were conventional. In green chili (green color), 12 samples were conventional and 12 were organic. The samples were collected from a controlled environment, and no vegetable presample preparation was involved before testing. In total, 10,800 spectral data were obtained from all three vegetables, and each individual vegetable (i.e., organic and conventional) constituted 1800 spectral data.

## 3. Results and Discussion

The hardware setup discussed in Section 2 collects spectral data with a 150 ms sampling rate of 6 data samples per second. The acquired spectral data are processed with the fuzzy engine to generate new features. The ACO algorithm analyzes all the generated features for optimal 12-channel feature selection. The selected 12-channel features are applied to the random forest algorithm and neural network model to classify organic and conventional vegetable samples. This results section is arranged as follows: Section 3.1 discusses the histogram analysis of 18-channel spectral data photon counts, followed by the NIR, UV and visible-band photon count distribution in Section 3.2. Section 3.3 describes the results of the ACO optimal channel feature selection mechanism. The performance of the random forest algorithm and neural network model classification is discussed in Section 3.4. The system’s response time and repeatability result analysis are discussed in Section 3.5 and Section 3.6.

### 3.1. Histogram Analysis of Photon Count Distribution–18 Channels

This section analyzes and visually illustrates the number of photons received by the multispectral sensor detector from the vegetable samples. Since the spectral response depends on the color of the vegetables, the histogram analysis was carried out for the three colored vegetable (red-color tomato, purple-color brinjal and green chili) samples taken for the analysis in the study at three different sections.

#### 3.1.1. Histogram Analysis of Photon Count Distribution to Discriminate Organic and Conventional Vegetable–Red Color Tomato

**Figure 4:**
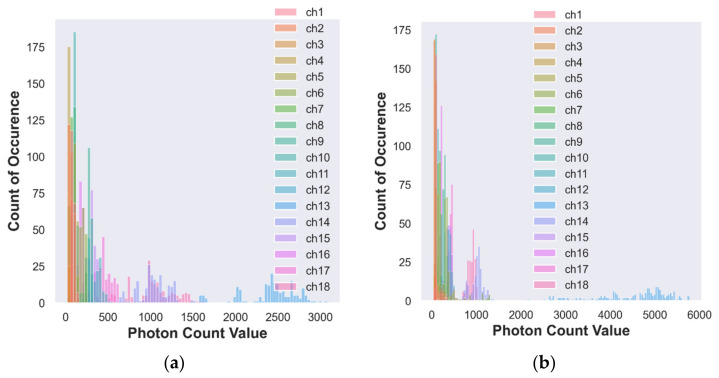
(**a**) Histogram of photon count for conventional tomato; (**b**) histogram of photon count for organic tomato.

The channel values can be distinguished by colors in the histogram. Figure 4a,b illustrates a few observations of the histogram analysis for conventional and organic tomato samples. Table 4 shows the maximum count of occurrence of the photon count that can be examined to find a peak-specific responsive band and channel for a given vegetable sample. The responses of photon count distribution to the constituent element present in the organic and conventional tomato.

In Channel 1, a photon count value of 128 is observed with a minimum count of occurrence of 2 times only and a maximum count of occurrence of 44 times for a photon count value of 66 for the conventional tomato sample. In the second column of Table 4, the first value of 2 refers to the minimum count of occurrence, and 128 represents the photon count value of the conventional tomato sample of Channel 1.

Likewise, a minimum count of occurrence of 2 times with a photon count value of 248 is recorded in Channel 1. A maximum count of occurrence of 69 times is observed with a 146 photon count value for the organic tomato sample. Similarly, the photon count values and count of occurrences for the remaining channels are listed in Table 4 for the conventional and organic tomato (red color) samples.

For conventional tomatoes, the maximum count of occurrence of 55 is recorded from Channel 13 (NIR, 730 nm) with a photon count value of 2300. The first minimum count of occurrence of 11 times with a photon count value of 68 is for Channel 3 (UV, 460 nm).

For organic tomatoes, the first maximum count of occurrence of 140 is recorded from Channel 6 (IR, 535 nm) with a photon count value of 300. The first minimum count of occurrence of 6 is in Channel 12 (Vis, 705 nm) with a photon count value of 102.

Table 4 shows that the conventional tomato of Channel 13 (NIR, 730 nm) obtains a maximum count of occurrence, and the organic tomato of Channel 6 (IR, 535 nm) also shows a maximum count of occurrence. Channels 13 and 6 are required to discriminate between the organic and conventional red-color tomato samples.

#### 3.1.2. Histogram Analysis of Photon Count Distribution to Discriminate Organic and Conventional Vegetable—Purple-Color Brinjal

**Figure 5:**
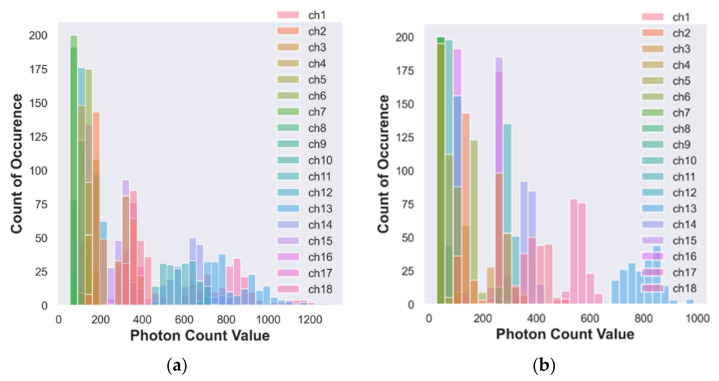
(**a**) Histogram of photon count for conventional brinjal; (**b**) histogram of photon count for organic brinjal.

Figure 5a,b visually illustrates a few key observations of the histogram analysis for conventional and organic brinjal samples.

In Table 5, Channel 1, a photon count value of 321 is observed with a minimum count of occurrence of 6 times for conventional brinjal.

From Table 5, the first maximum count of occurrence of 59 is recorded from Channel 13 (NIR, 730 nm) with a photon count value of 686, followed by an initial minimum count of occurrence of 9 times with a photon count value of 390 for Channel 3 (UV, 460 nm) obtained for the conventional brinjal sample.

Table 5 shows that the first maximum count of occurrence of 91 is observed from Channel 7 (Vis, 560 nm) with a photon count value of 31. The first minimum count of 7 occurred in Channel 12 (Vis, 705 nm) for a 342 photon count value for the organic brinjal sample.

Table 5 shows that the conventional brinjal sample shows a maximum count of occurrence at Channels 7 (Vis, 560 nm) and 13 (NIR, 730 nm) with a value of 59. Among Channels 7 and 13, the photon count reflection is high in Channel 13 (NIR, 730 nm); therefore, the Channel 13 (NIR, 730 nm) response provides a better response analysis for the conventional case of Channel 7 (Vis, 560 nm), which shows a maximum occurrence count for the organic brinjal channel. Differentiating between the conventional and organic brinjal samples of Channels 13 (NIR, 730 nm) and 7 (Vis, 560 nm) is required.

#### 3.1.3. Histogram Analysis of Photon Count Distribution to Discriminate Organic and Conventional Vegetable—Green-Color Chili

**Figure 6:**
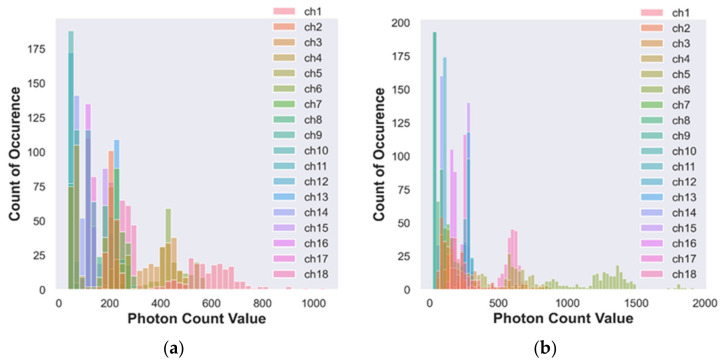
(**a**) Histogram of photon count for conventional Green chili; (**b**) histogram of photon count for organic green chili.

Figure 6a,b illustrates the histogram analysis of the conventional and organic chili sample histogram analysis.

Table 6 records a 1048 photon count value for a minimum count of 2 times from Channel 1 for conventional chili. A photon count value of 421 is recorded for a single count of occurrence of organic chili.

Table 6 shows that for the histogram analysis value for conventional green chili, the first maximum count of 91 is observed from Channel 4 (UV, 485 nm) with a photon count value of 50. Next, the minimum occurrence count of 10 is observed with a photon count value of 59 in Channel 9 (NIR, 610 nm).

For organic green chili, a starting maximum count of occurrence of 86 is observed from Channel 2 (UV, 435 nm) and a photon count value of 72. Next, a minimum count of occurrence of 7 is recorded in Channel 4 (UV, 485 nm) with a photon count value of 181.

Table 6 shows that for conventional green chili, the occurrence count is maximum in Channel 4 (UV, 485 nm), and for organic chili, Channel 2 (UV, 435 nm) shows a maximum count of occurrence. Therefore, it is concluded that Channels 4 and 2 show a better response to discriminate between the organic and conventional green chili samples.

Table 4, Table 5 and Table 6 show that to discriminate between the vegetable samples of red tomato, Channels 13 (NIR, 730 nm) and 6 (IR, 535 nm) are required. For purple-color brinjal, Channels 13 (NIR, 730 nm) and 7 (Vis, 560 nm), and for green chili, Channels 4 (UV, 485 nm) and 2 (UV, 485 nm), are required.

Each vegetable sample shows a maximum count of responses at different bands for discrimination.

Figure 4a,b, Figure 5a,b and Figure 6a,b depict most of the channels’ data are occupied on the exact count of occurrence of photon values. Therefore, it is tedious to distinguish between the photon count distribution for the individual channels due to overlapping on the same point. However, it is possible to analyze and locate the peak response band of the organic and conventional samples using the maximum count of occurrence.

### 3.2. Individual Channel Responses

In our proposed multispectral sensor system design, there are 18 channels available, divided into 3 prominent spectral bands, namely ultraviolet, visible and near-infrared, each consisting of 6 channels. The following section illustrates the NIR, UV and Vis spectral responses for organic and conventional brinjal samples. Each band shows better discrimination concerning the spectral photon count distribution for the brinjal sample. The individual-band spectral channel response is analyzed only for the purple-color brinjal sample alone as an example of illustration to reduce the number of pages in the article.

#### 3.2.1. UV Channel Response for Brinjal

**Figure 7:**
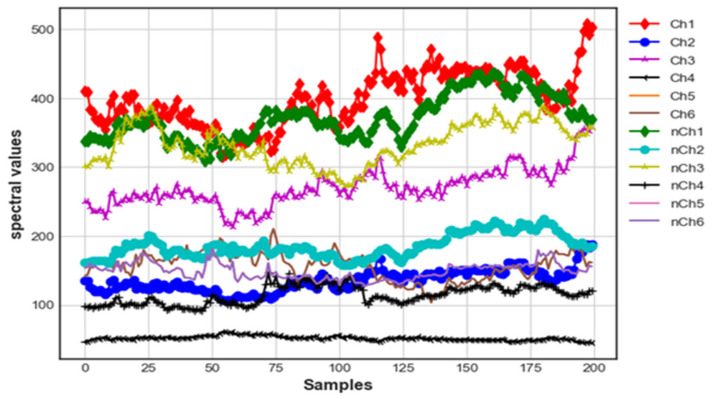
UV photon count response of organic and conventional brinjal samples.

Figure 7 depicts the samples collected from the 410–535 nm band with subwavelength bands of 410 nm, 435 nm, 460 nm, 485 nm, 510 nm and 535 nm. The wavelength-band amplitude levels for the organic and conventional brinjal samples of Channels 1, 3, 5 and 6 (i.e., 410 nm, 460 nm, 510 nm and 535 nm) are merged in most of the regions. It is hard to discriminate between the components. The wavelength-band amplitude levels of Channels 2 and 4 (435 nm and 485 nm) show better discrimination between the amplitude levels of organic and conventional components.

#### 3.2.2. NIR-VIS Channel Response for Brinjal

**Figure 8:**
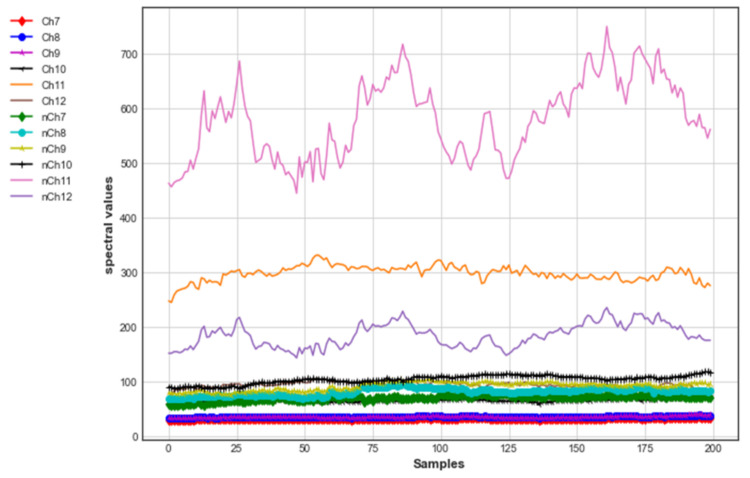
NIR-VIS (560–705 nm) photon count response of organic and conventional brinjal samples.

Figure 8 depicts the samples collected from the 560 to 705 nm bands with wavelengths of 560 nm, 585 nm, 610 nm, 680 nm and 705 nm on Channels 7, 8, 9, 11 and 12. Channels 7, 8, 9, 11 and 12 show better discrimination between the organic and conventional brinjal samples. Channel 10 (Vis, 645 nm) illustrates nondiscrimination between the organic and conventional brinjal samples because the amplitude levels collided.

**Figure 9:**
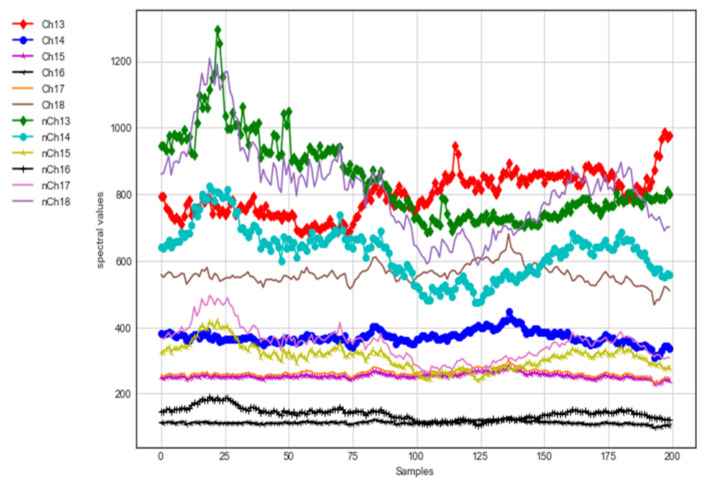
NIR-VIS (730–940 nm) photon count response of organic and conventional brinjal samples.

Figure 9 depicts the samples collected from the 730 to 940 nm band with wavelengths of 730 nm, 760 nm, 810 nm, 860 nm, 900 nm and 940 nm on Channels 13, 14, 15, 16, 17 and 18. Channels 13, 14 and 18 (730 nm, 760 nm and 940 nm) show better discrimination in the amplitude levels of the organic than conventional brinjal samples when compared to Channels 15, 16 and 17 (810 nm, 860 nm and 900 nm).

### 3.3. ACO Results

**Figure 10:**
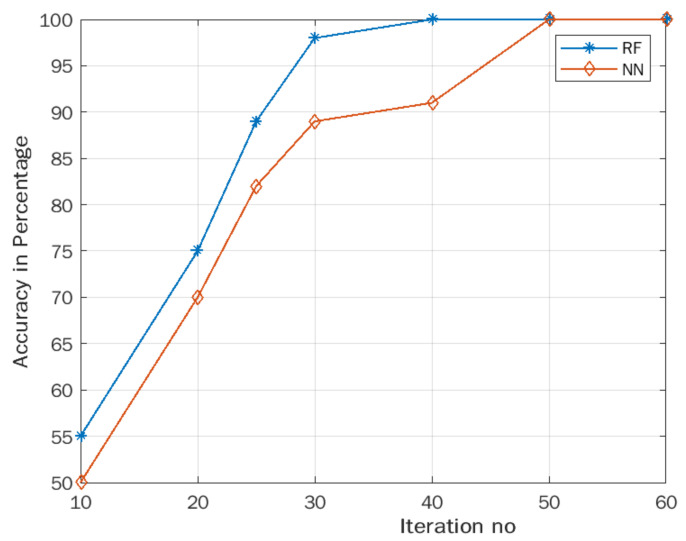
Accuracy vs. iteration number.

The analysis performed in Section 3.2 of individual band responses proves that only a few bands respond well, and many band data do not respond to discriminate between organic and conventional vegetable samples. Therefore, optimal feature selection is required to select the optimal band that responds better. To select such optimal features, the ACO algorithm was employed. The selected 12 optimal feature sets were applied to the random forest and neural network classifier. The results are shown in Figure 10. From Figure 10, it is seen that the accuracy of the random forest and neural network models is 100% and 90% at the 40th iteration and then converges to 100%. Convergence of RF and NN was observed at the 40th and 50th iterations. The ACO optimization algorithm is essential in optimizing and improving classification accuracy by optimal feature selection.

The optimized features were applied to the random forest classifier for organic and conventional vegetable sample discrimination. Optimally selected features (Sf_d_) were given to the random forest algorithm, and a classification accuracy of 100% was achieved. Table 7 compares the accuracy of the random forest and neural network models before and after ACO optimization for the organic and conventional vegetable samples.

### 3.4. Analysis of Neural Network Model

The ACO-selected optimal feature set was applied to the neural network model. The network was trained with 70% of the sample data. The validation phase use 15% of the sample data. Finally, the testing phase takes left out 15% of the sample data.

**Figure 11:**
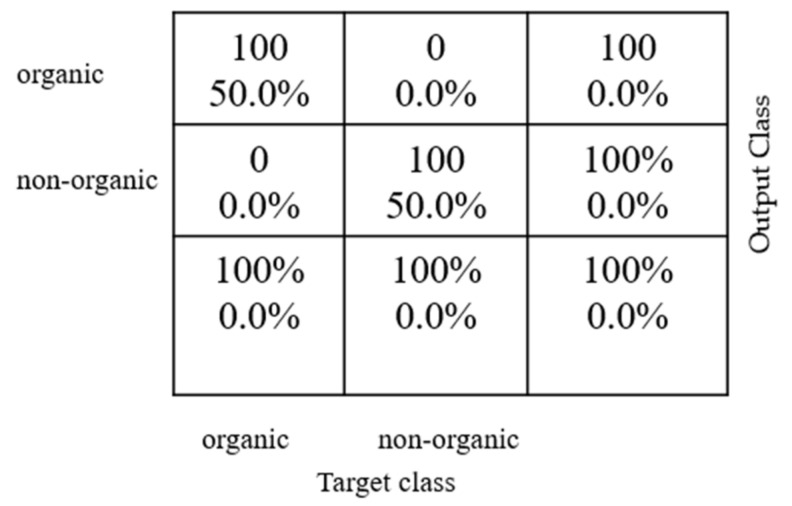
Confusion matrix for binary class of vegetable samples for neural network model.

Figure 11 depicts the predicted values, represented in the column, and the actual values, represented in the row. The diagonal axis represents the true-positive and true-negative labels classified for conventional and organic vegetables. The resultant classification achieves 100% accuracy in discriminating between conventional and organic samples.

**Figure 12:**
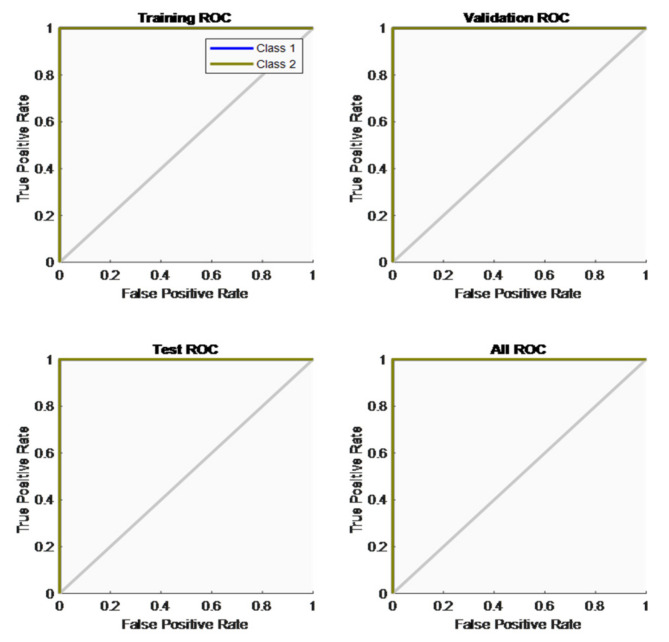
Receiver operating curve.

Figure 12 shows the receiver operating curve for the training, validation, test and all data sets. The receiver operating curve (ROC) was calculated for the true-positive rate (TPR) and false-positive rate (FPR), i.e., sensitivity and ROC between the false-positive rate (FPR) and false-negative rate (FNR), i.e., specificity. The ROC curve on the top left corner proves that the system works better with a performance of 100% accuracy for two-class classification models.

A comparison of the proposed model performance with similar literature is presented in Table 8.

In comparison with the similar literature work listed in Table 8, our proposed system performs better in the following aspects:

- The model proposed in [6] achieves an accuracy of 97.3% for organic and conventional yam discrimination. The cost of the experimented system model is expensive and not portable; therefore, mass spectroscopy is not applicable for real-time analysis. The system model is analyzed only with a single vegetable. The proposed work involves designing and constructing a nondestructive, less expensive, portable, rapid-analysis multispectral sensor system. It can discriminate between organic and conventional vegetables (tomato, green chili, brinjal) using fuzzy and ACO optimization algorithms for optimal feature selection. The optimal feature set is applied through random forest and neural network algorithms, achieving 100% classification accuracy.

Since one of our objectives is to reduce the cost and make it portable, the Triad multispectral sensor system was utilized for discrimination between organic and conventional vegetables. The Triad multispectral sensor costs around Rs. 6250/-. The cost of Raspberry Pi is around Rs. 1600/-. The ESP8266 Wi-Fi module costs around 1850/-. The completely designed equipment is much cheaper at Rs. 9700/-.

### 3.5. System’s Response Time

The response time of the entire sensor system was analyzed, and it was observed that the system can respond by taking a maximum time of 400 ms.

### 3.6. Repeatability Analysis

The repeatability response of the proposed system was tested at regular intervals over three months. Each time, three tests were carried out with different vegetable samples obtained from different markets (conventional samples). Organic samples were cultivated, taken from different soil places, and tested for the repeatability test. From the above repeatability test, it is observed that the system can reproduce the same results.

## 4. Conclusions

This research showed the potential usage of a multispectral sensor system for the nondestructive, rapid discrimination of organic and conventional green chili, tomato and brinjal. The proposed multispectral sensor is portable, which enables the consumer to efficiently use it in the field. The histogram analysis of 18-channel spectral responses provided discrimination between organic and conventional vegetable samples. Three spectral-band responses were plotted, depicting the discrimination between organic and conventional purple brinjal samples. The 18-channel raw data were tested with a neural network and random forest algorithm and obtained 89% and 92% classification accuracy. The classification accuracy was improved to 100% by the new fuzzy feature set generation and optimal feature set selected by the ACO algorithm. The organic and conventional discrimination results are displayed on the developed web page through the IoT application system. Authenticated users can access the detected output anywhere with the developed system. The future scope can be enhanced by testing other vegetable samples grown under different soil conditions. Vegetable samples grown under the use of DAP fertilizer were tested, which can be extended by testing with other similar types of fertilizers. Pesticide- and insecticide-sprayed vegetable sample testing can also be expanded. Some chemicals are sprayed on vegetables to look fresh, and this kind of toxication can be identified in the future for storage purposes. Sample testing in the proposed work was performed in a controlled environment. On, farm field testing can be explored in the near future.

## Figures and Tables

**Table 1:** Spectroscopic analysis methods for organic and conventional fruits/vegetables discrimination.

Fruits/Vegetables	Spectroscopic Analysis and Methodology Applied	Maximum Accuracy
Olives [5]	NIR and PLS-DA	85.9%
Fuji apple [9]	NIR and LSVM	98%
Apple [10]	NIR and KNN, SVM, PLS-DA	94%
Gala Apples [11]	NIR and PLS-DA	96–98%
Tomato [12]	^1^H NMR, MIR, IRMS and PCA, LDA, PLS-DA	100%
Pineapple [13]	Digital Refractometer and KNN, PCA, LDA, PLSR, MSC-PCA-LDA	100%

**Table 2:** Input triangle membership function a,b,c points.

Input/Band	Input Crisp Value Range	Membership Function Value [a, b, c]
Channel [1–6] NIR band	Range (1–308–360) L, Range (361–395) M, Range (395–436) H,	[1, 308, 334], [361, 378, 395], [415.5, 436]
	Range(1–105–130) L, Range (130–155) M, Range (155–224) H	[1, 105, 117.5], [130, 142.5, 155], [189.5, 224]
	Range (1–212–271) L, Range (271–380) M, Range (380–557) H	[1, 212, 241.5], [271, 325.5, 380], [468.5, 557]
	Range (1–45–50) L, Range (50–60) M, Range (60–148) H	[1, 45, 47.5], [50, 55, 60], [104, 148]
	Range (1–81–91) L, Range (91–107) M, Range (107–210) H	[1, 81, 86], [91, 99, 107], [158.5, 210]
	Range(1–103–150)L, Range (150–180) M, Range (180–210) H	[1, 103, 126.5], [150, 165, 180], [180, 195]
Channel [9,11,13–16] Visible band	Range (1–31–33) L, Range (33–38) M, Range (38–103) H,	[1, 31, 32], [33, 35.5, 38], [70.5, 103]
	Range (1–244–300) L, Range (300–329) M, Range (329–750) H	[1, 244, 272], [300, 314.5, 329], [539.5, 750]
	Range (1–680–840) L, Range (840–986) M, Range (986–1295) H	[1, 680, 760], [840, 913, 986], [1140.5, 1295]
	Range (1–31–400) L, Range (400–447) M, Range (447–825) H	[1, 31, 215.5], [400, 423.5, 447], [636, 825]
	Range (1–238–250) L, Range (250–284) M, Range (284–420) H	[1, 238, 244], [250, 267, 284], [352, 420]
	Range (1–97–112) L, Range (112–129) M, Range (129–192) H	[1, 97, 104.5], [112, 120.5, 129], [160.5, 192]
Channel [7,8,10,12,17,18] UV band	Range (1–25–30) L, Range (30–33) M, Range (33–97) H	[1, 25, 27.5], [30, 31.5, 33], [65, 97]
	Range (1–30–34) L, Range (34–37) M, Range (37–95) H	[1, 30, 32], [34, 35.5, 37], [66, 95]
	Range (1–58–63) L, Range (63–71) M, Range (71–120) H	[1, 58, 60.5], [63, 67, 71], [95.5, 120]
	Range (1–76–92) L, Range (92–103) M, Range (103–245) H	[1, 76, 84], [92, 97.5, 103], [174, 245]
	Range (1–230–259) L, Range (259–99) M, Range (299–495) H	[1, 230, 244.5], [259, 279, 299], [397, 495]
	Range (1–467–558) L, Range (558–679) M, Range (78–148) H	[1, 467, 512.5], [558, 618.5, 679], [348, 458]

**Table 3:** Output membership functions key port.

Output Membership Function	Points [a, b, c]
Very Low	[1, 2, 2]
Low	[2, 4, 4]
Medium	[4, 6, 6]
High	[5, 8, 8]

**Table 4:** Eighteen-channel photon count distribution and its number of occurrences for conventional and organic tomato.

Channel No	Minimum Count of Occurrence— Photon Count		Maximum Count of Occurrence— Photon Count	
	Conventional	Organic	Conventional	Organic
1	2-128	2-248	44-66	69-146
2	4-77	1-80	44-46	51-113
3	11-68	2-216	37-76	68-152
4	3-32	1-105	31-44	56-88
5	2-76	1-250	46-125	96-160
6	2-139	1-525	46-123	140-300
7	1-42	1-285	54-61	58-95
8	5-68	2-376	50-95	60-174
9	3-335	1-482	42-271	91-256
10	2-114	1-158	53-98	53-112
11	2-221	2-275	52-258	39-301
12	1-76	6-102	42-86	37-102
13	1-1820	1-2048	55-2300	62-4834
14	2-621	1-391	40-925	92-992
15	1-212	1-172	48-295	63-368
16	1-141	1-83	48-181	81-211
17	6-254	1-119	52-425	84-413
18	4-913	1-328	52-913	80-836

**Table 5:** Eighteen-channel photon count distribution and its number of occurrences for conventional and organic brinjal.

Channel No	Minimum Count of Occurrence— Photon Count		Maximum Count of Occurrence—Photon Count	
	Conventional	Organic	Conventional	Organic
1	6-321	1-488	41-334	41-430
2	6-197	2-179	38- 184	41-122
3	9-390	7-342	32-308	58-256
4	4-147	3-61	32-97	50-50
5	1-210	2-105	43-149	53-84
6	3-181	1-113	39-154	52-151
7	6-57	1-34	59-70	91-31
8	5-95	4-38	46-81	51-35
9	5-89	1-40	39-95	52-34
10	2-95	3-70	40-105	59-64
11	1-750	1-261	33-505	59-296
12	3-235	4-101	29-152	68-93
13	2-1295	1-956	59-686	45-834
14	4-755	1-434	47-650	66-365
15	3-366	1-285	58-311	66-251
16	5-165	1-331	56-140	87-111
17	3-425	1-244	55-355	66-258
18	1-1022	1-659	47-835	74-552

**Table 6:** Eighteen-channel photon count distribution and the number of occurrences for conventional and organic green chili.

Channel No	Minimum Count of Occurrence— Photon Count		Maximum Count of Occurrence—Photon Count	
	Conventional	Organic	Conventional	Organic
1	2-1048	1-421	52-496	63-143
2	1-175	3-310	63-194	86-72
3	2-308	5-545	37-438	84-126
4	1-175	7-181	91-50	49-64
5	1-368	2-518	82-185	44-577
6	1-466	3-1805	1-466	50-1236
7	1-309	5-196	1-309	37-167
8	6-160	1-116	31-179	56-63
9	10-59	3-24	42-63	43-25
10	2-63	1-40	43-52	76-33
11	1-165	2-317	66-114	41-274
12	1-72	5-122	62-49	53-106
13	5-288	1-319	61-122	65-281
14	5-89	3-73	49-72	53-78
15	1-297	3-305	71-193	49-280
16	1-172	5-161	74-121	50-168
17	1-164	3-291	52-128	48-260
18	1-383	5-686	57-170	40-606

**Table 7:** Comparison of ACO accuracy.

Binary Classification	Before ACO RF/NN	After ACO RF/NN
Organic and conventional vegetable discrimination	92/88	100/100

**Table 8:** Performance comparison.

Literature Work for Organic and Conventional Discrimination	Type of Spectroscopy Employed	Methodology Employed	Fruits/Vegetables Tested	Accuracy Achieved
Lyu, C., et al. [7]	Mass spectroscopy	Random forest classifier	Chinese yam	97.3%
Proposed work with 18-channel raw data	Multispectral sensor system	Binary classification Neural network/random forest classifier	Green chili, tomato and brinjal	89%/92%
Proposed work with fuzzy feature generation and ant colony optimization algorithm	Multispectral sensor system	Binary classification Neural network/random forest classifier	Green chili, tomato and brinjal	100%

## Data Availability

The data presented in this study are available on request from the corresponding author. The data are not publicly available due to confidentiality.
